# Complete genome sequence of *Staphylococcus hominis* FSEL1 isolated from chicken skin in South Korea

**DOI:** 10.1128/mra.00243-24

**Published:** 2024-06-11

**Authors:** Ga-Hee Ban

**Affiliations:** 1 Department of Food Science and Biotechnology, Ewha Womans University, Seoul, South Korea; University of Maryland School of Medicine, Baltimore, Maryland, USA

**Keywords:** *Staphylococcus hominis*, chicken skin, whole-genome sequencing

## Abstract

*Staphylococcus hominis* FSEL1 has been isolated from chicken skin. The complete genome sequence of the bacterium comprised one chromosome and two plasmids of 2.44 Mbp. A total of 2,273 CDS, 2,473 genes, 19 rRNAs, 62 tRNAs, and 1 tmRNA were predicted to be present within the genome.

## ANNOUNCEMENT

Although numerous *Staphylococcus* species have been previously isolated from chickens (1–3), many studies have placed an emphasis on *Staphylococcus aureus*. The recent emergence of non-*aureus* staphylococci (NAS) as opportunistic pathogens with antimicrobial resistance (AMR) has led to increased investigation of AMR gene prevalence in *Staphylococcus* species (4, 5). Here, the complete genome sequence of *S. hominis* FSEL1, isolated from chicken skin and exhibiting tetracycline resistance, is reported.

The raw chicken skin was purchased from a grocery store in Seoul, South Korea in February 2023, and a 25 g sample was enriched in 225 mL of tryptic soy broth (TSB) at 37°C for 24 h. The Baird Parker medium selects for coagulase-positive bacteria including *S. aureus* and NAS. One such colony was enriched and was identified as *S. hominis* using an API staph kit and a single isolated colony was grown in TSB until the exponential phase. Whole-genome sequencing was conducted to explore the genomic components and to enhance our knowledge of AMR prevalence in NAS.

Genomic DNA was extracted using the HiGene genomic DNA prep kit (Biofact, Korea). The sequencing was performed using Oxford Nanopore technology. Libraries were prepared with 500 ng of genomic DNA using a ligation sequencing kit (SQK-LSK109, Oxford Nanopore Technologies, UK) without shearing and size selection, according to the manufacturer’s instructions. Sequencing was then carried out using a Flongle (R9.4.1) flow cell on the Oxford Nanopore MinION instrument for 24 h. Base calling and demultiplexing were performed using Guppy version 6.4.6 using the high-accuracy model. The raw data comprised 160,776 reads and an average read quality of 12.1. NanoPlot version 1.30.1 (6) was used for quality control of the raw nanopore reads. Low-quality reads were trimmed using Filtlong version 0.2.1. The top 95% of reads based on nucleotide quality scores were retained, and reads shorter than 1,000 bp were discarded. *De novo* assembly and its circularization were conducted using Trycycler version 0.5.4 (7). The final assembly was polished using Medaka version 1.7.2 (github.com/nanoporetech/medaka) until a consensus was derived, and the quality was assessed using QUAST version 5.0.2 (8) and BUSCO version 5.2.2 (9). Circlator version 1.5.1 was then used to circularize the corrected assembly and align the bacterial genome with dnaA as the origin (10). The final consensus sequence revealed 97.1% of complete BUSCOs and 1.6% of fragmented BUSCOs within the bacteria odb10 data set. The complete genome of *S. hominis* FSEL1 comprised of one chromosome and two plasmids with a GC content of 31.46%, and a coverage depth of 339×. The plasmids measured 27,953 bp and 26,704 bp, with GC contents of 30% and 30.5%, respectively. Genome annotation was conducted using NCBI Prokaryotic Genome Annotation Pipeline (11), yielding 2,273 coding sequences, 2,473 genes.

Screening of contigs for AMR and virulence genes was conducted using ABRicate version 0.7 (12) and is presented in Table 1. One virulence gene and three AMR genes were identified. All software employed in the current study utilized default parameters, except where otherwise noted.

**TABLE 1:** Screening of antimicrobial and virulence genes of *S. hominis* FSEL1 using ABRicate

Gene	Product description	% coverage	% identity	Start	End	Database	Accession
clpP	ATP-dependent Clp endopeptidase proteolytic subunit	94.3	78.23	1,968,359	1,968,922	vfdb	NP_465991
fusC	Fusidic acid resistance EF-G-binding protein gene family	100	100	50,132	50,770	ncbi	NG_050413
tet(K)	Tetracycline efflux MFS transporter	100	100	570	1,949	ncbi	NG_048200
qacB	Subunit of the qac multidrug efflux pump	100	99.94	26,080	27,624	ncbi	NG_048038

## Data Availability

The complete genome sequence of *S. hominis* FSEL1 has been deposited in GenBank under the accession number, CP121263-CP121265. The associated BioProject and BioSample accession numbers are PRJNA951503 and SAMN34044562, respectively.
